# RE: Positive models of suffering and psychiatry

**DOI:** 10.1192/bjb.2024.25

**Published:** 2024-06

**Authors:** Om Prakash

**Affiliations:** Professor of Psychiatry, Geriatric Mental Health Division, Institute of Human Behaviour and Allied Sciences, New Delhi, India. Email: drjhirwalop@yahoo.co.in

*Integrating positive and negative models of suffering: a proposal for a unified approach in psychiatric practice*

I was prompted by the recent publication in the *BJPsych Bulletin* by Huda, ‘Positive models of suffering and psychiatry’,^1^ to express my views regarding the juxtaposition of negative and positive models of suffering within psychiatric practice. Although Huda provides a nuanced discussion on the traditional approach to alleviating suffering versus a perspective that sees potential value in suffering, the delineation offers a critical reflection yet also suggests a potential area of confusion for both practitioners and patients. The discourse sets a foundational understanding that whereas the alleviation of suffering is a cornerstone of medical practice, as echoed in the ethos of clinical epidemiology,^2^ there exists a parallel narrative that suffering may serve as a conduit for personal growth and enlightenment, aligning with broader existential and psychological theories.^3,4^ This dichotomy, although enriching, may inadvertently complicate the therapeutic landscape, suggesting a necessity for a more integrated approach that harmonises these models to enhance patient care. Accordingly, I propose the consideration and development of a unified model that assimilates the ethical imperative to mitigate suffering with a recognition of the transformative potential inherent in the experience of suffering. This model would aim to: (a) prioritise the immediate and compassionate alleviation of suffering as a primary objective of psychiatric intervention, in line with traditional medical practice;^2^ (b) acknowledge the potential for suffering to catalyse personal growth, transformation and the acquisition of new perspectives, as detailed in the literature on post-traumatic growth;^3^ (c) empower patients by involving them in treatment decisions, echoing the principles of narrative medicine and patient-centred care;^5^ (d) foster treatment flexibility, recognising the individual's unique experience of suffering and the dynamic nature of their needs and potential for growth.^4^ Such a unified model proposes a more holistic and nuanced approach to psychiatric care, one that not only seeks to alleviate pain but also respects the complex, multifaceted nature of human suffering. The implementation of this model would necessitate a shift towards a more integrative psychiatric education and practice, one that values the depth of human experience as much as the alleviation of symptoms. The dialogue initiated by Huda is invaluable, and it is within this context that I propose a further exploration of how we, as a psychiatric community, can better integrate these models to serve our patients. This endeavour would not only clarify our therapeutic objectives but also potentially enrich psychiatric practice with a deeper understanding and respect for the intricacies of the human condition.
